# Accumulation of an Antidepressant in Vesiculogenic Membranes of Yeast Cells Triggers Autophagy

**DOI:** 10.1371/journal.pone.0034024

**Published:** 2012-04-18

**Authors:** Jingqiu Chen, Daniel Korostyshevsky, Sean Lee, Ethan O. Perlstein

**Affiliations:** Lewis-Sigler Institute for Integrative Genomics, Princeton University, Princeton, New Jersey, United States of America; Chiba University Center for Forensic Mental Health, Japan

## Abstract

Many antidepressants are cationic amphipaths, which spontaneously accumulate in natural or reconstituted membranes in the absence of their specific protein targets. However, the clinical relevance of cellular membrane accumulation by antidepressants in the human brain is unknown and hotly debated. Here we take a novel, evolutionarily informed approach to studying the effects of the selective-serotonin reuptake inhibitor sertraline/Zoloft® on cell physiology in the model eukaryote *Saccharomyces cerevisiae* (budding yeast), which lacks a serotonin transporter entirely. We biochemically and pharmacologically characterized cellular uptake and subcellular distribution of radiolabeled sertraline, and in parallel performed a quantitative ultrastructural analysis of organellar membrane homeostasis in untreated vs. sertraline-treated cells. These experiments have revealed that sertraline enters yeast cells and then reshapes vesiculogenic membranes by a complex process. Internalization of the neutral species proceeds by simple diffusion, is accelerated by proton motive forces generated by the vacuolar H^+^-ATPase, but is counteracted by energy-dependent xenobiotic efflux pumps. At equilibrium, a small fraction (10–15%) of reprotonated sertraline is soluble while the bulk (90–85%) partitions into organellar membranes by adsorption to interfacial anionic sites or by intercalation into the hydrophobic phase of the bilayer. Asymmetric accumulation of sertraline in vesiculogenic membranes leads to local membrane curvature stresses that trigger an adaptive autophagic response. In mutants with altered clathrin function, this adaptive response is associated with increased lipid droplet formation. Our data not only support the notion of a serotonin transporter-independent component of antidepressant function, but also enable a conceptual framework for characterizing the physiological states associated with chronic but not acute antidepressant administration in a model eukaryote.

## Introduction

Cationic amphiphilic/amphipathic drugs (CAD) represent a subset of Food and Drug Administration (FDA) approved compounds that promiscuously interact with both proteinaceous and non-proteinaceous targets, the latter being cellular membranes [1], [2]. CAD association with cellular membranes depends on an ionizable amine that is positively charged at physiological pH and a lipophilic polycyclic scaffold, but does not depend on stereochemistry, as in the peculiar case of the antidepressant sertraline/Zoloft® moonlighting as a fungicide [3]. The primary protein target of sertraline is thought to be the human serotonin transporter (hSERT), which localizes to synaptic clefts and recycles the monoamine neurotransmitter serotonin after each burst of neurotransmission. According to the monoamine hypothesis of depression, antidepressants like sertraline bind hSERT and acutely block reuptake of serotonin in the brain [4]. However, a latency period whose molecular basis is unknown precedes the emergence of the actual antidepressant effect in humans, and in rodent behavorial models of depression, suggesting that antidepressants exert additional effects at targets besides hSERT. Given the well known and wide-ranging effects of CAD on cellular membrane homeostasis in the absence of specific proteins targets [5], [6], the clinical relevance of antidepressant accumulation in neuronal cell membranes has been vigorously debated. For example, there is evidence that supports the existence of serotonin transporter-independent components of antidepressant function in vertebrate cellular models [7], some of which appears to involve membrane accumulation by antidepressants [8], [9]. Yet a comprehensive model of antidepressant function that accounts for all drug-target interactions in the human brain has so far been elusive.

The goal of the present study is to begin developing and validating a comprehensive model of complex antidepressant function in humans. The first step in this arduous process is to reconcile two pharmacological perspectives that have historically dominated conventional thinking about CAD activity in cells lacking specific integral membrane protein targets. On the one hand, a molecular view of drug-membrane interactions derives from the seminal work of Singer and Sheetz on amphipath-induced morphological transformations of freshly isolated human erythrocytes, a cell-based model system superior to reconstituted liposomes but still lacking endomembranes. Singer and Sheetz proposed the bilayer couple/balance model, which states that a charged amphipath preferentially accumulates at equilibrium in the leaflet (monolayer) exhibiting the opposite net charge [10]. A disparity in inter-leaflet surface area of less than 1% resulting from asymmetric partitioning by charged amphipaths can be readily observed as dramatic macroscopic changes in the topology of the erythrocyte plasma membrane. On the other hand, a physiological view was developed around the same time by Christian de Duve and colleagues, and is called lysosomotropism, or “ion trapping.” Lysosomotropism is defined as the concentrative capacity of acidic organelles to trap protonated weak bases within, and cannot be modeled by red blood cells [11]. Lysosomotropism has been documented in various mammalian cell lines and in whole organisms treated with CAD.

Here we build on an effort begun in our previous study of sertraline-induced “overdose” [12], in which we demonstrated that the model eukaryote *Saccharomyces cerevisiae* (budding yeast) is an ideal experimental system in which to combine the biophysical insights of the bilayer couple model with the physiological insights of lysosomotropism. In that study, we reported the isolation and genetic characterization of sertraline overdose-resistant mutants (sert^R^) with altered clathrin function or reduced vacuolar H+-ATPase complex activity. Others have also shown that yeast is amenable to studying cellular membrane accumulation by CAD [13]–[15]. However, a caveat of our previous study is that selection for (sert^R^) mutants required supra-therapeutic (∼10^−5^ M) drug concentrations. Here we applied techniques of classical pharmacology to yeast, which enabled us to measure membrane accumulation by radiolabeled sertraline – hereafter [^3^H]sertraline – at clinically relevant (∼10^−9^ M) concentrations. We conclude the present study by proposing an evolutionarily informed model of antidepressant function that may provide a molecular basis for neurotrophism induced by chronic treatment with antidepressants in rodent models of human depression, and by extension the therapeutic lag observed in patients taking antidepressants.

## Results

### Two thermodynamic drivers of sertraline entry into yeast cells

**Figure 1:**
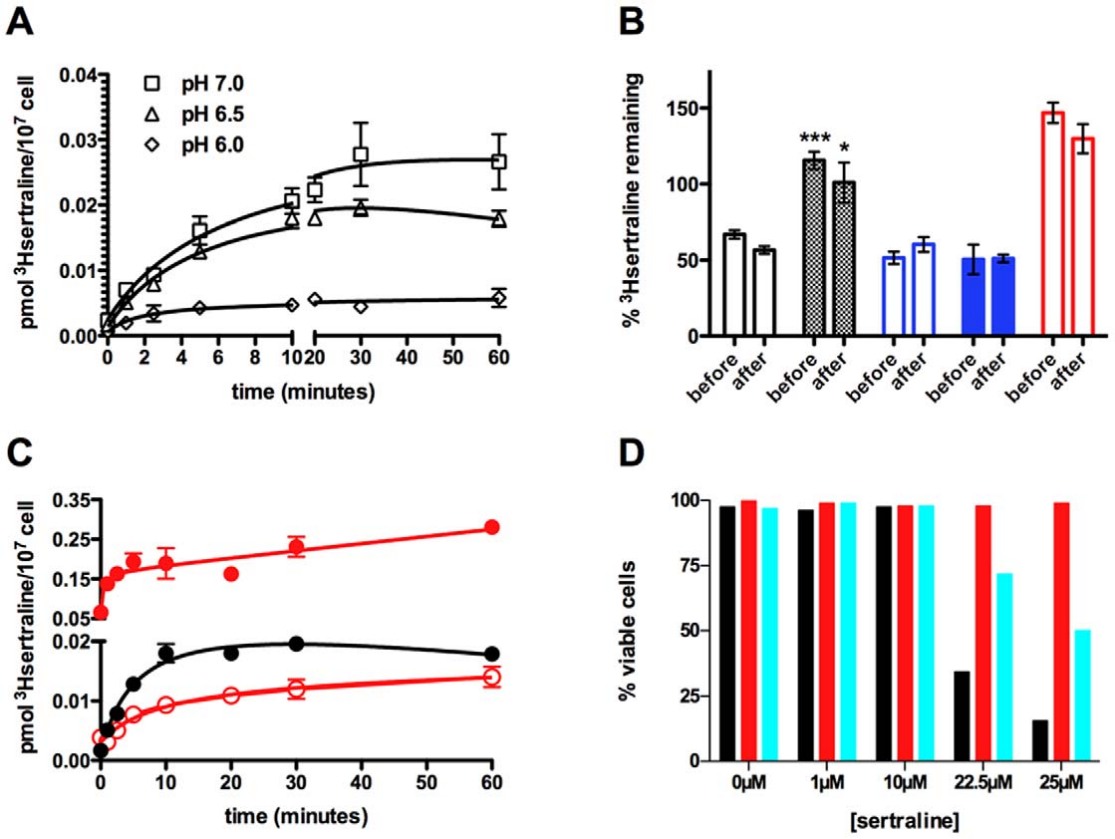
[^3^H]sertraline cellular uptake and accumulation is rapid, partially lysosomotropic and varies from cell to cell. (**A**) Wildtype BY4716 cells accumulate [^3^H]sertraline (picomoles of radioligand/10^7^cells) in a time-dependent manner as a function of ambient pH: pH 6.0 (diamonds); pH 6.5 (triangles); pH 7.0 (squares). Notice the break in the x-axis between 10 and 20. (**B**) Bar graph showing fraction of [^3^H]sertraline accumulated with a 30-minute 2 µM bafilomycin A pre-treatment (“before”) compared to no bafilomycin A pre-treatment; and fraction of [^3^H]sertraline accumulated with a 30-minute 2 µM bafilomycin A-post-treatment (“after”) compared to no bafilomycin A post-treatment. Wildtype BY4716 (black, unfilled) is contrasted with a *vma9* mutant (black, filled). Before and after treatments of wildtype BY4716 treated with 1 µM FCCP (blue, unfilled), 1 µM FCCP+2 µM bafilomycin A (blue, filled), or 10 µg/mL oligomycin (red, unfilled) are also shown. Statistical significance determined by two-tailed t-test. *** P = 0.0004; * P = 0.0118. (**C**) [^3^H]sertraline uptake and accumulation time course with wildtype BY4742 cells (black circles, filled) a *dnf1Δ dnf2Δ dnf3Δ* triple mutant cells (red circles, filled), and a *vma9* mutant (red circles, unfilled). Notice the break in the y-axis between 0.02 and 0.05. (**D**) Single-cell dose response of sertraline-induced cytotoxicity (“overdose”) for wildtype BY4716 (black), *vma9* (red), and *cup5* (cyan) cells. Error bars indicate SEM. Means were generated from three independent biological replicates.

We treated wildtype BY4716 cells (hereafter “wildtype) with [^3^H]sertraline, which we obtained by custom synthesis (see Materials and Methods). We report a total [^3^H]sertraline cellular accumulation (B_max_) equal to 0.019 picomoles (pmol) per 10^7^ cells (+/−0.0014 SEM), and a half-maximal [^3^H]sertraline cellular uptake rate equal to 3.1 minutes (+/−0.97 SEM) (**Fig. 1A**). These data are consistent with the lysosomotropic mechanism originally described de Duve and colleagues [11]. Briefly, as the pH of the extracellular medium increases, the deprotonation of sertraline is favored; the ratio of neutral to cationic species reaches unity at the pKa of sertraline. Neutral sertraline is membrane-permeable while charged sertraline is not. Therefore, more [^3^H]sertraline is internalized by cells growing in alkaline media compared to acidic media. Several classical studies showed that cellular uptake and accumulation of radiolabeled tricyclic antidepressants by primary neurons and fibroblast cell lines is lysosomotropic and Na^+^-independent [16], [17].

However, [^3^H]sertraline cellular uptake is only partially dependent on proton motive forces generated by vacuolar H^+^-ATPase complexes (V-ATPases) [18], which can be specifically inhibited by the macrolide antibiotic bafilomycin A (BAF). Pre-treatment of wildtype cells with BAF for 30 minutes resulted in a 65% reduction in [^3^H]sertraline cellular accumulation compared to the control condition, while treatment of wildtype cells with BAF 30 minutes *after* exposure to [^3^H]sertraline resulted in reduced [^3^H]sertraline cellular accumulation that was 57% of the control amount (**Fig. 1B**). We performed four controls in order to demonstrate the specificity of V-ATPase-dependent proton motive forces. First, a *dnf1,2,3*Δ triple mutant, which exhibits constitutive vacuolar hyper-acidification [19], significantly hyper-accumulates [^3^H]sertraline while the *vma9* mutant, which exhibits constitutive vacuolar alkalinization, hypo-accumulates [^3^H]sertraline (**Fig. 1C**). Second, the effects of BAF on [^3^H]sertraline accumulation are completely abolished in a *vma9* (*YCL005W-A*) mutant, which normally encodes subunit e of the V0 subunit of the V-ATPase complex (**Fig. 1B**). Third, before and after treatments of wildtype cells with oligomycin, a specific chemical inhibitor of the F1-F0 mitochondrial ATPase, actually resulted in slightly increased [^3^H]sertraline accumulation (**Fig. 1B**). Fourth, FCCP, a non-specific proton ionophore, phenocopies the effects BAF but co-administration of these two agents does not exhibit additivity (**Fig. 1B**). Interestingly, single cells overdose in the presence of sertraline in a stochastic manner (**Fig. 1D**). Thus, at the population level and at the level of single cells, the cellular uptake of [^3^H]sertraline appears to be non-uniform; a fraction of internalized sertraline is “ion trapped,” while the remainder is associated with cellular membrane sites.

**Figure 2:**
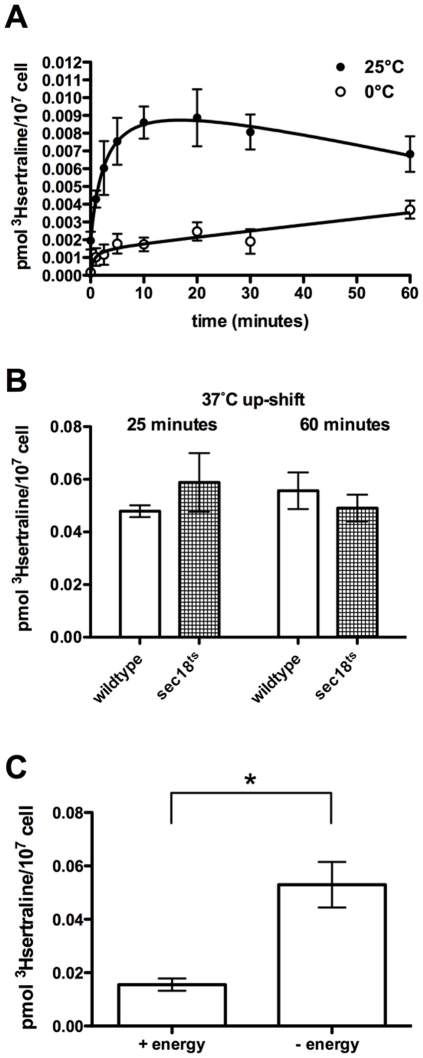
Solubility properties of cellular membranes affect the rate and extent of [^3^H]sertraline accumulation. (**A**) One-hour time course of [^3^H]sertraline accumulation in BY4716 cells incubated at 25°C (black circles, filled), wildtype incubated at 0°C (black circles, unfilled). Low temperature values were normalized by room temperatures values for ease of comparison. (**B**) [^3^H]sertraline accumulation was measured in wildtype BY4742 cells (unfilled) and *sec18^ts^* mutant cells (hatches). Times indicate duration of radioligand exposure at 37°C. (**C**) Total [^3^H]sertraline accumulation in the presence and absence of energy poisons. Statistical significance determined by two-tailed t-test. * P = 0.0113. Error bars indicate SEM. Means were generated from three independent biological replicates (except for (**B**), which was generated from two independent biological replicates).

Next we measured [^3^H]sertraline cellular uptake and accumulation in response to several environmental perturbations that affect cellular membrane function globally. First, we examined the effect of low temperature, as low temperature promotes the liquid crystalline-gel transition of membranes, i.e., decreases membrane fluidity. Membrane fluidity has been shown to be a determinant of local anesthetic partitioning into reconstituted liposomes [20]. The initial rate of [^3^H]sertraline cellular uptake by wildtype cells is four times slower at 0°C versus 25°C; after 60 minutes, cells incubated at 0°C accumulate 45% of the total [^3^H]sertraline taken up by isogenic cells incubated at 25°C (**Fig. 2A**). To rule out that low temperature mediates this dampening effect through cessation of vesicle-mediated transport, we also measured [^3^H]sertraline cellular accumulation by a *sec18^ts^* mutant, which is conditionally unable to perform membrane-membrane fusion reactions after temperature up-shift [21]. We observed no significant differences between *sec18^ts^* and its wildtype reference after a short (25 minute) or long (60 minute) incubation at the non-permissive temperature (**Fig. 2B**). Next, we tested whether [^3^H]sertraline cellular uptake and accumulation depends on energy. We pretreated wildtype cells with a cellular ATP depleting cocktail containing 10 mM sodium azide and 10 mM 2-deoxy-D-glucose. Total [^3^H]sertraline cellular accumulation was increased 3.4-fold in the presence of energy poisons (**Fig. 2C**). We interpret this result to mean that energy-dependent xenobiotic efflux pumps constitutively extrude [^3^H]sertraline from the cell. Thus, the association of [^3^H]sertraline with cellular membranes appears to depend on bulk physical properties of the bilayer.

### Sertraline permeates vesiculogenic membranes

**Figure 3:**
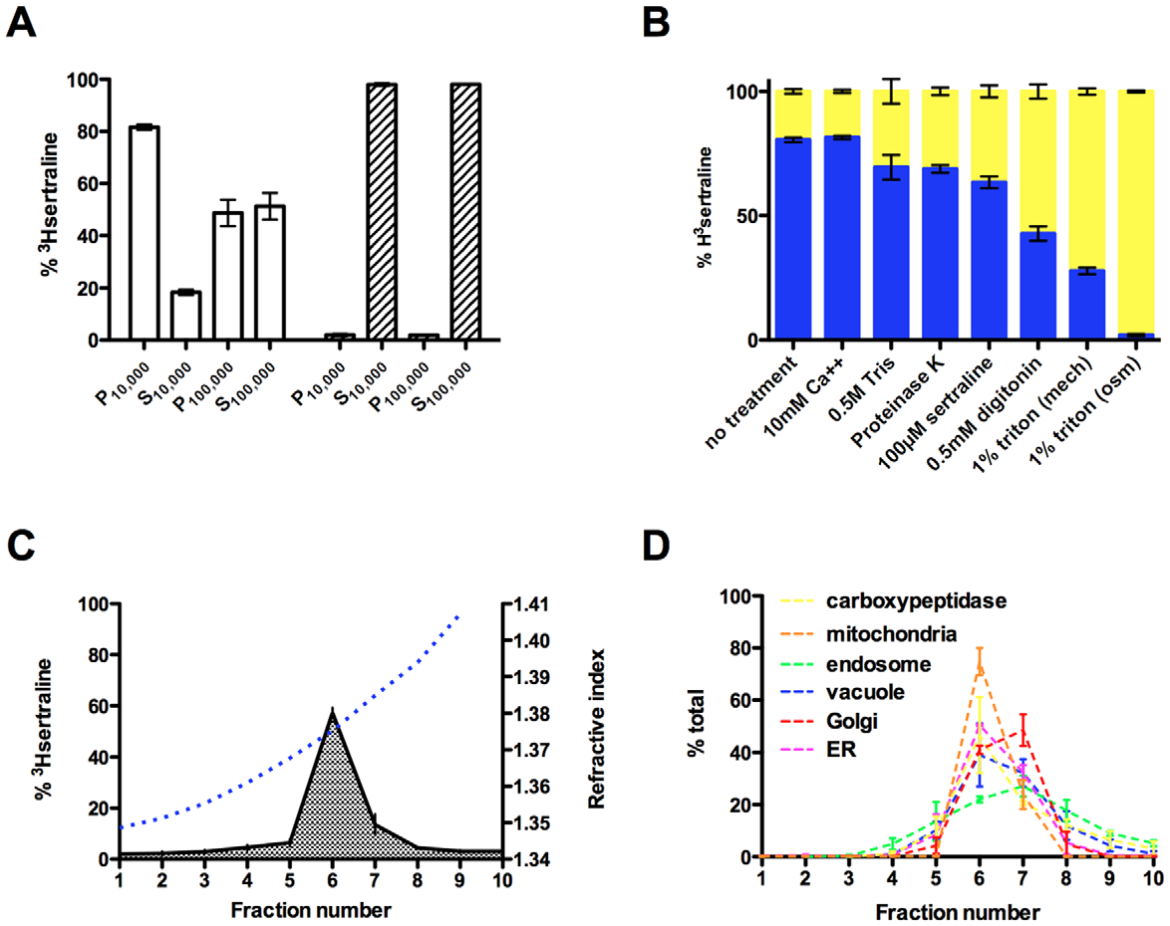
Subcellular fractionation demonstrates [^3^H]sertraline accumulation in vesiculogenic membranes. (**A**) The percent [^3^H]sertraline detected in soluble (“S”) versus pellet (“P”) fractions following sequential centrifugations (10,000×*g* and 100,000×*g*) in the absence (white columns) or presence of 0.5% Triton X-100 detergent (hatches). (**B**) The ratio of soluble (yellow) to P_10,000_-associated (blue) [^3^H]sertraline following extraction with a diverse panel of chemical agents. * P<0.05, ANOVA; ** P<0.0001, ANOVA. (**C**) Distribution of [^3^H]sertraline across ten equal volume fractions following Optiprep gradient separation of total organellar membranes (P_100,000_) from wildtype BY4716 cells treated with [^3^H]sertraline for one hour at 25°C. The refractive index is plotted (blue slashed line) on the left y-axis. (**D**) Densitometry plots of six organellar markers distributed across the ten gradient fractions. Error bars indicate SEM. Means were generated from three independent biological replicates.

We characterized the subcellular distribution of [^3^H]sertraline in wildtype cells by biochemical fractionation experiments. As shown in **Figure 3A**, over 80% of intracellular [^3^H]sertraline sediments in the P_10,000_ fraction following osmotic lysis; that percentage climbs to 90% after mechanical lysis (**Fig. 3B**). After accounting for the trace amount of [^3^H]sertraline present in the P_100,000_ microsomal fraction, only 10–15% of intracellular [^3^H]sertraline appears to be truly soluble, demonstrating that at equilibrium the vast majority of [^3^H]sertraline stably partitions into cellular membranes, presumably as a neutral species. To verify that [^3^H]sertraline partitioned into cellular membranes we performed the same experiment but in the presence of the nonionic detergent Triton X-100. Triton X-100 completely solubilizes P_10,000_-associated [^3^H]sertraline (P<0.0001, ANOVA; **Fig. 3A**). However, detergent-sensitive cellular membrane binding sites may not be chemically uniform. We reasoned that we could distinguish between at least two types of membrane association – adsorption (topical) versus intercalation (deep) – on the basis of chemical extractability of [^3^H]sertraline from the P_10,000_ fraction. The results of this analysis are presented in **Figure 3B**. Chemical agents that disrupt electrostatic interactions (e.g., 0.5 M Tris) either have no or little solubilizing effect on membrane-associated [^3^H]sertraline, while chemical agents that disrupt hydrophobic interactions, including the mild detergent digitonin, solubilize membrane-associated [^3^H]sertraline to varying degrees. Proteolytic digestion of surface-exposed membrane proteins has a modest solubilizing effect, ruling out membrane proteins as essential for amphipath-membrane association. Interestingly, excess (100 µM) “cold” sertraline only has a modest solubilizing effect, which is consistent with the notion that sertraline is buried in the hydrophobic phase of the bilayer at equilibrium.

A P_10,000_ fraction is thought to be enriched in large organelles like vacuoles and mitochondria at the expense of small organelles like Golgi and ER. As an initial attempt to biochemically purify sertraline-associated cellular membranes, we performed analytical density-gradient centrifugation on a P_100,000_ fraction after a single-step centrifugation of lysates from wildtype cells. A single peak spanning two fractions of intermediate density (refractive index in the range 1.37–1.38) contains on average ∼70% of membrane-associated [^3^H]sertraline, with ∼60% concentrated in a single fraction (fraction 6) (**Fig. 3C**). We screened these gradient fractions against a panel of antibodies specific for markers residing in different subcellular compartments, and densitometry plots are shown for each marker in **Figure 3D**. Although our gradient had limited resolving power at fractions 6–7, we observed the strongest co-localization of markers specific for ER and vacuolar membrane markers, as well as the vacuolar resident enzyme carboxypeptidase, with the [^3^H]sertraline peak in fraction 6, but we did not observe co-localization with a plasma membrane marker (data not shown). We observed less albeit still significant co-localization of Golgi and endosomal membrane markers with [^3^H]sertraline, though these markers themselves peak in the slightly denser fraction 7. Although the mitochondrial marker porin is also present in fractions 6–7, we showed above that disrupting proton motive forces with oligomycin actually increased [^3^H]sertraline accumulation (**Fig. 1B**), so while association with mitochondrial cannot be ruled out it appears coincidental.

### Pharmacological characterization of mutants with altered sertraline sensitivity

We reasoned that comparison of measurements of [^3^H]sertraline cellular uptake and accumulation in a panel of mutant strains with altered sertraline cellular response would allow us to develop a cell biological model of cellular membrane accumulation by sertraline. The wildtype strain serves as a reference for three previously described *de novo* sert^R^ mutants: *vma9* (described above), *swa2* (*YDR320C*) and *chc1* (*YGL206C*) [12]. ACY769 is a wildtype prototrophic strain derived from S288c (hereafter “prototroph”), which serves as a reference for four sertraline-hypersensitive (sert^HS^) homozygous gene deletion mutants involved in clathrin coat formation: *arf1*Δ, *cdc50*Δ, *drs2*Δ and *sac1*Δ. *ARF1* (*YDL192W*) encodes a small GTPase that is thought to be a master regulator of vesiculogenesis at internal membranes. *DRS2* (*YAL026C*) encodes an aminophospholipid flippase that localizes to Golgi and endosomal membranes, and *CDC50* (*YCR094W*) encodes its regulatory subunit. *SAC1* (*YKL212W*) encodes a lipid phosphatase with specific activity against phosphatidylinositol 4-phosphate (PIP), and localizes to the endoplasmic reticulum (ER) and Golgi. Together these sert^R^ and sert^HS^ mutants comprise a “vesiculogenesis” module centered around *ARF1* and PIP, as deletion of *ARF1* is synthetically lethal with loss of *SWA2*, *DRS2* or *CDC50* [22], [23]. Total [^3^H]sertraline cellular accumulations (B_max_) for each of the mutants appear in **Table 1**.

**Table 1:** Total [^3^H]sertraline cellular accumulation (B_max_) as a function of pH. B_max_ is defined as pmol/10^7^ cells/hour.

		B_max_	B_max_ 95% CI	r^2^
	**BY4716**	0.00558 (+/−0.000532)	0.00448 to 0.00669	0.593
	***chc1***	0.0199 (+/−0.00288)**	0.0139 to 0.0259	0.403
	***swa2***	0.0118 (+/−0.00127)**	0.00919 to 0.0144	0.457
	***vma9***	-	-	-
**pH 6.0**	**ACY769**	0.0109 (+/−0.000767)	0.00933 to 0.0124	0.522
	***drs2Δ***	0.0166 (+/−0.000805)**^ns^**	0.0149 to 0.0183	0.881
	***cdc50Δ***	0.0183 (+/−0.00114)**^ns^**	0.0159 to 0.0206	0.844
	***arf1Δ***	0.0168 (+/−0.00223)**^ns^**	0.0122 to 0.0215	0.462
	***sac1Δ***	0.0399 (+/−0.00250)**	0.0343 to 0.0447	0.826

		B_max_	B_max_ 95% CI	r^2^
	**BY4716**	0.0189 (+/−0.00140)	0.0161 to 0.0217	0.575
	***chc1***	0.0363 (+/−0.00591)*	0.0240 to 0.0486	0.333
	***swa2***	0.0196 (+/−0.00245)**^ns^**	0.0145 to 0.0247	0.499
	***vma9***	0.0142 (+/−0.00111)	0.0119 to 0.0165	0.750
**pH 6.5**	**ACY769**	0.0326 (+/−0.00136)	0.0299 to 0.0353	0.864
	***drs2Δ***	0.0401 (+/−0.00224)**^ns^**	0.0355 to 0.0447	0.873
	***cdc50Δ***	0.0410 (+/−0.00295)**^ns^**	0.0348 to 0.0471	0.835
	***arf1Δ***	0.0747 (+/−0.00688)*	0.0605 to 0.0890	0.750
	***sac1Δ***	0.0896 (+/−0.00857)**	0.0718 to 0.107	0.685

		B_max_	B_max_ 95% CI	r^2^
	**BY4716**	0.0292 (+/−0.00217)	0.0248 to 0.0337	0.826
	***chc1***	0.0750 (+/−0.00633)**	0.0619 to 0.0882	0.682
	***swa2***	0.0797 (+/−0.00533)**	0.0686 to 0.0907	0.847
	***vma9***	-	-	-
**pH 7.0**	**ACY769**	0.0639 (+/−0.00203)	0.0599 to 0.0680	0.925
	***drs2Δ***	0.0724 (+/−0.00460)**^ns^**	0.0628 to 0.0819	0.900
	***cdc50Δ***	0.0717 (+/−0.00516)**^ns^**	0.0610 to 0.0824	0.860
	***arf1Δ***	0.128 (+/−0.0159)**^ns^**	0.0946 to 0.161	0.715
	***sac1Δ***	0.246 (+/−0.0148)**	0.215 to 0.277	0.877

Unlike the *vma9* mutant or BAF-treated wildtype cells, the *chc1* mutant and to a lesser extent the *swa2* mutant, unexpectedly hyper-accumulate [^3^H]sertraline. Therefore it may be more appropriate to classify *chc1* and *swa2* mutants as “sertraline-tolerant.” This hyper-accumulation is distinct from that exhibited by *dnf1,2,3*Δ, as both *chc1* and *swa2* have a constitutive vacuolar acidification defect that phenocopies V-ATPase deficiency [12]. Controlling for cell number and cell size, we observed that two other mutants hyper-accumulate [^3^H]sertraline: *sac1*Δ and *arf1*Δ. *drs2*Δ and *cdc50*Δ mutants are indistinguishable from the control condition. These results indicate that there are at least two qualitatively distinct phases of the cellular response to sertraline: the first phase affects acute sertraline uptake, while the second phase, which is typified by *drs2*Δ, has no effect on acute sertraline uptake, and so instead must be involved in the adaptation to chronic sertraline exposure.

**Figure 4:**
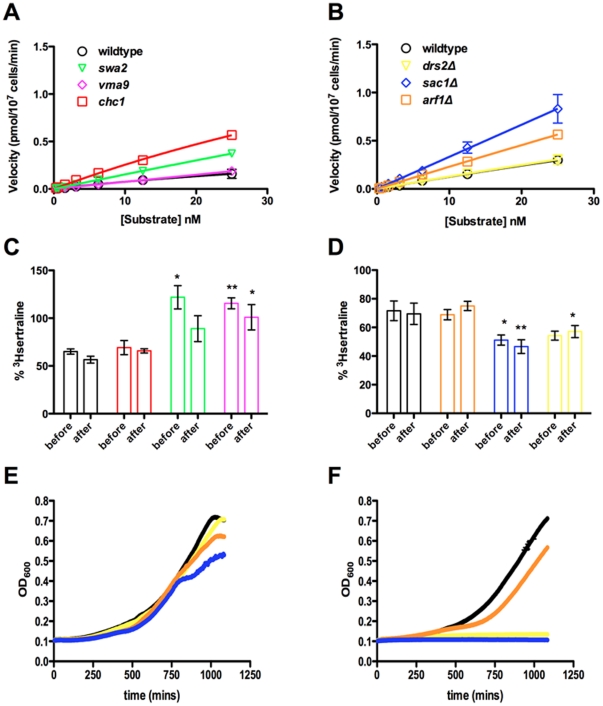
Altered kinetics of [^3^H]sertraline cellular uptake in sertraline-resistant (sert^R^) and sertraline-hypersensitive (sert^HS^) mutants. (**A**) Kinetic analysis of [^3^H]sertraline cellular uptake after two minutes for sert^R^ mutants (*chc1*, *swa2* and *vma9*). (**B**) Kinetic analysis of [^3^H]sertraline cellular uptake after two minutes for sert^HS^ mutants (*arf1*Δ, *drs2*Δ and *sac1*Δ). The concentrations of [^3^H]sertraline tested were 0.3125 nM, 0.625 nM, 1.563 nM, 3.125 nM, 6.25 nM, 12.5 nM, and 25 nM. Wildtype reference strains are plotted as black unfilled circles. Strains are identified by a fixed color scheme. Before and after treatments with bafilomycin A are shown for sert^R^ (**C**) and sert^HS^ (**D**) strains. Multi-hour growth curve for wildtype and thre sertraline-hypersensitive mutants. Growth in the absence (**E**) and in 20 µM sertraline (**F**) are shown. Statistical significance determined by two-tailed t-test: * P<0.05; ** P<0.01. Fits were generated by nonlinear regression. Error bars indicate SEM. Means were generated from three independent biological replicates.

To explore this phenomenon more rigorously, we measured the kinetics of [^3^H]sertraline cellular uptake as a function of substrate concentration. Kinetic experiments with both wildtype references and mutants with altered sertraline cellular response indicate a single non-saturable component of [^3^H]sertraline cellular uptake, i.e., internalization by simple diffusion (**Fig. 4A–B**). We estimate V_max_ to be 0.62 pmol [^3^H]sertraline/10^7^ cells/min, which translates to ∼1000 sertraline molecules per cell per minute subject, of course, to individual differences between clones. We estimate the K_m_ of sertraline to be 71.5 nM, which represents high-affinity binding but less than the affinity exhibited by it for the human serotonin transporter [24]. The panel of mutants exhibited a range of acute [^3^H]sertraline cellular uptake rates. Neither *vma9* nor *drs2*Δ mutants show a measurable difference in the rate of [^3^H]sertraline cellular uptake compared to wildtype. However, as expected *chc1*, *swa2*, *arf1*Δ and *sac1*Δ take up more [^3^H]sertraline per unit time, in the order *sac1Δ>chc1* = *arf1*Δ>*swa2*. Acute sertraline hyper-accumulation appears to have two different molecular bases, as indicated by the results of screening the mutants on a secondary phenotype of BAF-induced release of internalized [^3^H]sertraline (**Fig. 4C–D**).

Among the sert^R^ mutants, *swa2* is more resistant to the effects of BAF than wildtype, while *chc1* is unchanged compared to wildtype, indicating that one component of sertraline's cellular membrane association may be complexly regulated by the rate of clathrin assembly and disassembly. The *sac1*Δ mutant appears to accumulate sertraline by a completely distinct mechanism. *sac1*Δ cells exhibit a near 20% reduction in the fraction of [^3^H]sertraline retained after inhibition of V-ATPase complexes compared to wildtype (**Fig. 4D**). This result suggests that another component of sertraline's cellular membrane association may be regulated by the levels of the phosphoinositide PIP. Phosphoinositides have been shown to constitute anionic binding sites for CAD at the interfacial region of the bilayer in addition to the phosphate groups forming the backbone of glycerophospholipids [25]. We also observed that cell growth rate in the presence of overdose concentrations of sertraline is not correlated with the rate of nanomolar [^3^H]sertraline cellular uptake, as clearly demonstrated by a comparison between the sert^HS^ mutants *sac1*Δ and *drs2*Δ (**Fig. 4E–F**). This result demonstrates that phospholipid asymmetry, as opposed to membrane anionicity per se, is a key component of the adaptive physiological response to chronic cellular membrane accumulation by sertraline, as was originally proposed by Huestis and colleagues in experiments on erythrocytes [26].

### Ultrastructural analysis of autophagy induction and membrane quality control

**Figure 5:**
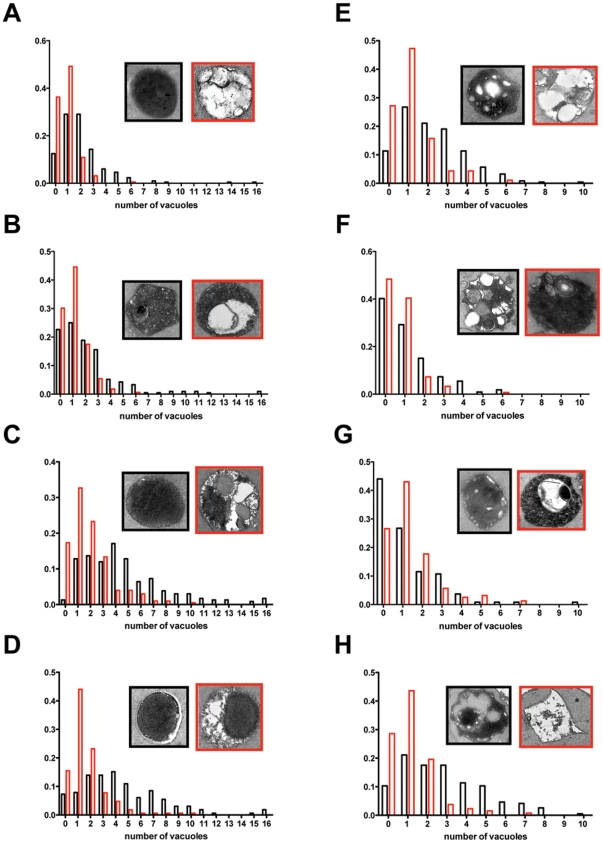
Vacuolar number, morphology and contents assessed from transmission electron micrographs of untreated and sertraline-treated cells. (**A**) BY4716; (**B**) *vma9*; (**C**) *swa2*; (**D**) *chc1*; (**E**) prototroph; (**F**) *sac1*Δ; (**G**) *drs2*Δ; (**H**) *arf1*Δ. For each strain, the distribution of vacuoles per cell is shown for a population of untreated cells (black columns) and 60 µM sertraline-treated cells (red columns). Insets contain a representative vacuole from the untreated (black outline) and 60 µM sertraline-treated (red outline) populations.

The pharmacological and biochemical experiments described above localize sertraline to vesiculogenic membranes. We therefore performed an ultrastructural examination of organellar membrane homeostasis on yeast cells exposed to 60 µM sertraline for 45 minutes, which constitutes a sub-lethal chronic treatment (**Fig. S1**). If the bilayer couple model holds, then exogenous cationic amphipaths will favor the more negatively charged leaflet of organellar membranes. In the specific case of sertraline, we expected to find examples of damaging local curvature stress in membranes comprising the vesicular transport pathway. Initial support for this hypothesis was found in the observation that micromolar sertraline treatment has three noteworthy effects on vacuole homeostasis, which serves as a quantitative cell biological read out. First, we observed a decrease in the number vacuoles per cell in wildtype cells treated with 60 µM sertraline (**Fig. 5A**). The percentage of vacuole-less cells increases (36% vs. 12%) as does the percentage of cells containing one large consolidated vacuole (49% vs. 29%). In fact, loss of vacuoles was observed in all strains treated with 60 µM sertraline (**Fig. 5B–H**). Second, the steady-state distributions of vacuoles per cell are increased relative to wildtype in three mutants: *chc1*, *swa2* and *arf1*Δ. Interestingly, the vacuoles of the sert^HS^ mutant *arf1*Δ exhibits polygonal and tubulated morphologies (**Fig. 5H**), while the vacuoles of the sert^R^ mutants *chc1* and *swa2* by and large exhibit wildtype vacuolar morphology. However, the vacuoles of *chc1* and *swa2* cells are more likely to have regions of thickened bilayer (**Fig. 5D** **, inset**). The seminal study by Lieber *et al* documented membrane thickening in erythrocytes bathed in the phenothiazine chlorpromazine [27]. Third, we observed a sharp increase in the percentage of electron-lucent wildtype vacuoles (24% vs 97%) (**Fig. 5A** **, inset**), and comparable increases were observed across all the strains (**Fig. 5** **, red insets**).

We reasoned that the distribution of vacuoles per cell would allow us to test the hypothesis that sertraline hyper-accumulation is caused by an increase in the surface-area-to-volume ratio of vacuoles or other V-ATPase-acidified organelles of the vesicular transport pathway. The steady-state number of vacuoles per cell separates the four sertraline hyper-accumulating mutants into two unequal groups. Group One is *arf1*Δ, *chc1*, and *swa2*, three genes that interact physically and genetically [22]. Group Two is *sac1*Δ; *sac1*Δ cells accumulate the phosphoinositide PIP, which recruits and activates *ARF1* and presumably stimulates vesiculogenesis. Changes in the number or surface-area-to-volume ratio of organelles may explain the hyper-accumulation phenotype of Group One mutants, but does not appear to explain the hyper-accumulation phenotype of *sac1*Δ cells, which have fewer vacuoles per cell at steady state. Also, the vacuolar membranes of *sac1*Δ cells appear to have an aberrant crenated morphology (**Fig. 5F** **, inset**).

**Figure 6:**
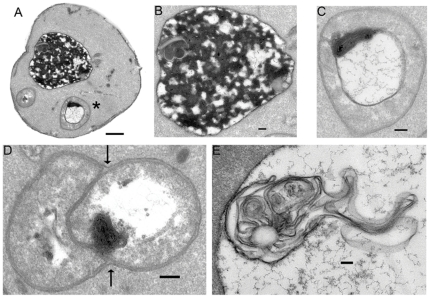
A time course of sertraline treatment reveals early and persistent induction of autophagy and the appearance of aberrant multilamellar structures. (**A**) Representative 60 µM sertraline-treated wildtype BY4716 cell after 5 minutes of sertraline treatment. Magnification is 6,300× (scale bar = 0.5 micron). The lone vacuole of the cell in (**A**) is shown at higher magnification (16,000×; scale bar = 0.1 micron) in panel (**B**). The black asterisk denotes an autophagosome that has encapsulated a membranous whorl, which is shown at higher magnification (25,000×; scale bar = 0.1 micron) in (**C**). The black arrows in (**D**) mark the telltale double membrane of an autophagosome at higher magnification (25,000×; scale bar = 0.1 micron). An elaborated multilamellar structure from a BY4716 cell treated with 60 µM sertraline for 20 minutes is shown at higher magnification (16,000×; scale bar = 0.1 micron) in (**E**).

The spike in electron lucency combined with a reduction in the total number of vacuoles per cell is accompanied by the appearance of double membrane-bound autophagosomes, a tell-tale sign of autophagy. A typical sertraline-treated wildtype cell after five minutes of exposure to 60 µM sertraline is illustrative (**Fig. 6A**). Changes in gross vacuolar morphology, electron lucency, and fine vacuolar membrane structure are typical of sertraline-treated cells at all time points, and a magnified view of a representative vacuole highlights the salient changes (**Fig. 6B**). The aberrant multilamellar membranous structure marked by an asterisk in **Figure 6A** is clearly encased in an autophagosome (**Fig. 6C**). Quantification of autophagosomes like those appearing in **Figure 6C–D** in wide-field images corroborates the single-cell snapshots: untreated wildtype cells contain on average 0.11 autophagosomes, while sertraline-treated cells contain 0.48, an increase of 4.2-fold. Multilamellar structures encapsulated by autophagosomes were observed in wildtype cells at all time points. Interestingly, similar membranous structures have been observed in brain tissues of rodents treated with the illicit psychoactive CAD 3,4-Methylenedioxymethamphetamine (MDMA) [28]. Two particularly striking examples are highlighted. In **Figure 6D**, black arrows mark the sites where two unilamellar vesicles, one of which contains a densely packed membranous whorl, are clearly discerned from the outer membrane of the autophagosome. And in **Figure 6E**, lamellar plumes of membrane form a Medusa-like structure trapped inside a large electron-lucent vacuole.

**Figure 7:**
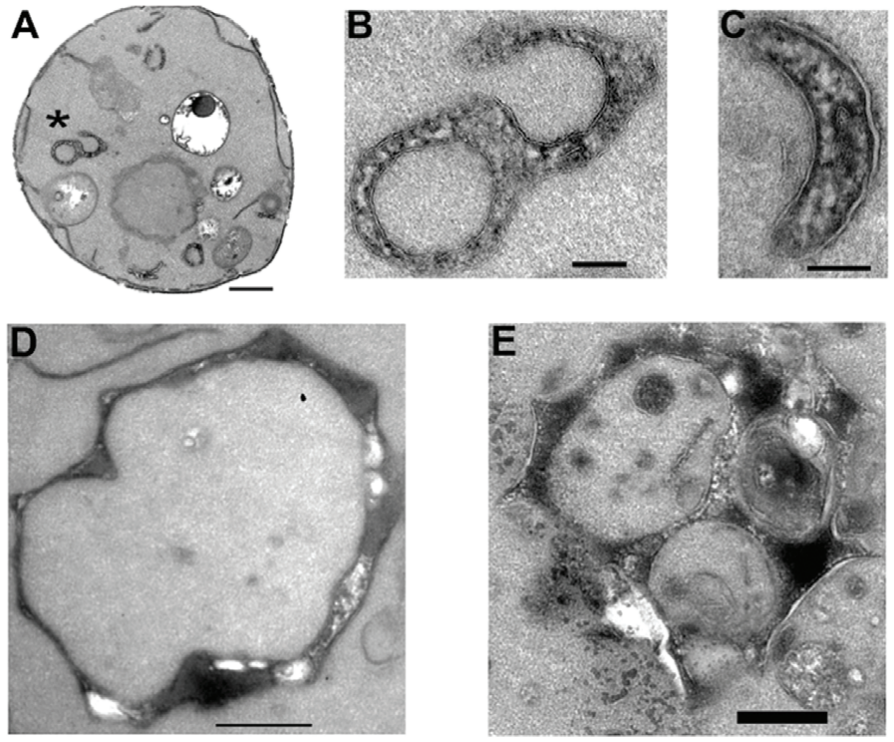
Ultrastructural evidence of local membrane curvature stress in sertraline-treated cells. (**A**) Representative 60 µM sertraline-treated wildtype BY4716 cell at 8,000× (scale bar = 0.5 micron). Asterisk denotes circularized Golgi-like structure, which is shown at higher magnification (31,500×; scale bar = 0.1 micron) in (**B**). An enlarged Golgi-like cisterna with visible expansion of the convex membrane from a 60 µM sertraline-treated wildtype prototroph cell at high magnification (40,000×; scale bar = 0.1 micron) is shown in (**C**). An irregular organelle with normal membrane thickness from a typical untreated *arf1*Δ cell at 8,000× (scale bar = 0.5 micron) is shown in (**D**). A comparable structure exhibiting localized membrane expansion in a 60 µM sertraline-treated *arf1*Δ cell at 8,000× (scale bar = 0.5 micron) is shown in (**E**).

If autophagy is triggered by sertraline accumulating in organellar membranes, one would expect to observe ultrastructural evidence of membrane curvature stress throughout the vesicular transport pathway, not just in vacuolar membranes. In this instance, a representative wildtype cell treated with 60 µM sertraline for 45 minutes is illustrative (**Fig. 7A**). The structure marked by an asterisk is a shown at higher magnification to be a dilated cisterna, possibly a Golgi stack or an autophagosomal precursor, with circular or crescent morphology (**Fig. 7B**). Similar structures were observed in other sertraline-treated cells at this time point, including an example of a dilated cisterna with clearly thickened membranes on the convex surface. (**Fig. 7C**). Comparison of an untreated *arf1*Δ cell to a sertraline-treated *arf1*Δ cell reveals several localized exaggerated regions of membrane expansion in comparable organellar structures (**Fig. 7D–E**).

**Figure 8:**
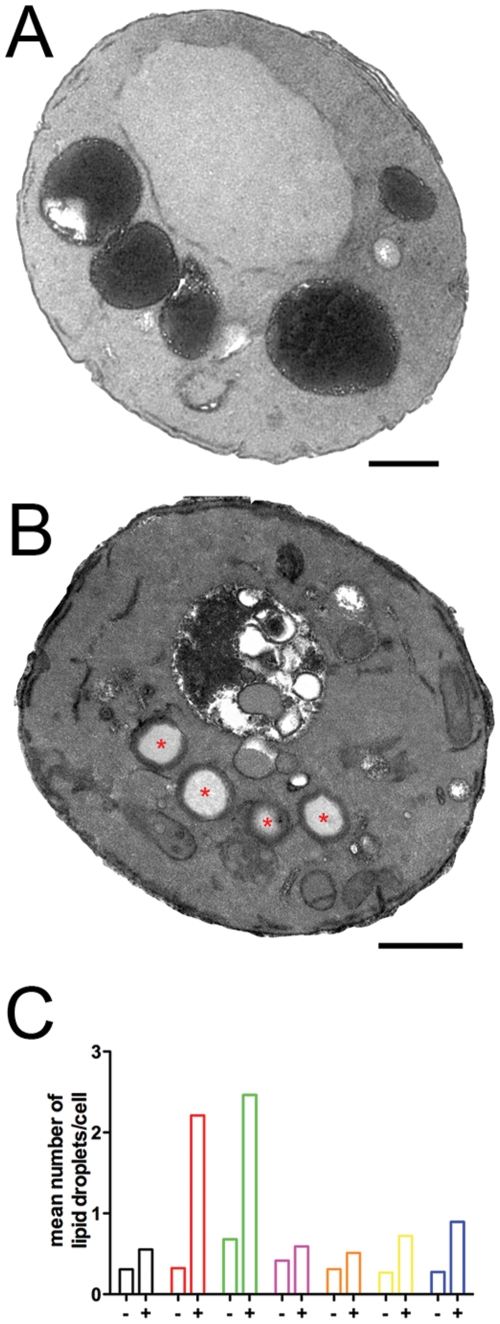
The *chc1* mutant exhibits unique adaptation to chronic sertraline exposure. (**A**) A representative untreated *chc1* cell contains five electron-dense vacuoles (10,000×; scale bar = 0.5 micron). (**B**) A representative sertraline-treated *chc1* cell contains a single vacuole and four lipid droplets marked by red asterisks (10,000×; scale bar = 0.5 micron). (**C**) Quantification of lipid droplet formation in untreated and sertraline-treated cells. The “-“ column correspond to lipid droplet counts performed on untreated cells. The “+” column correspond to lipid droplet counts performed on cells treated with 60 µM sertraline for 45 minutes. The strain identities are: BY4716 (black); *chc1* (red); *swa2* (green); *vma9* (magenta); *arf1*Δ (orange); *drs2*Δ (yellow); *sac1*Δ (blue).

Finally, two mutants, *chc1* and *swa2*, exhibited a unique ultrastructural phenotype that may be involved in adaptive cellular pathways that degrade and regenerate cellular membranes infiltrated by exogenous cationic amphipaths. At steady state, *chc1* and *swa2* mutants exhibit a vacuolar expansion/fragmentation phenotype that is “normalized” after 60 µM sertraline treatment for 45 minutes insofar as the number of vacuoles per cell decreases (**Fig. 5C–D**). However, these vacuoles exhibit a non-random distribution of osmiophilic lumenal contents and appear to contain undigested vesicular compartments (**Fig. 8B**). Interesting, the number of lipid droplets, which are storage depots for neutral lipids (e.g., triglycerides) and marked by red asterisks (**Fig. 8B**), increased after sertraline treatment in wildtype and sert^R^ and sert^HS^ mutant strains by an unknown mechanism (**Fig. 8C**). This increase is most pronounced in sertraline-treated *chc1* and *swa2* cells; untreated *chc1* cells have a mean lipid droplet count equal to 0.32, while sertraline-treated *chc1* cells have a mean lipid droplet count equal to 2.2. By contrast, untreated wildtype cells have a mean lipid droplet count equal to 0.31, while sertraline-treated wildtype cells have a lipid droplet count equal to 0.55. One explanation for this result is that lipid droplets form during adaptation of yeast cells to secretory pathway stress, as has been suggested by others [29].

## Discussion

We presented evidence that supports an evolutionarily informed, cell biological explanatory model of cellular membrane accumulation by sertraline in a simple eukaryote with an intact secretory pathway. Explication of our model begins with the passive internalization of neutral sertraline molecules at the plasma membrane. Lysosomotropism acts as an amplifier of simple diffusion, and suggests a mechanism whereby sertraline may distribute non-uniformly throughout vertebrate tissues as a result of tissue-specific activity or regulation of V-ATPase-dependent acidification [30]. The internalization of sertraline appears to follow an entry route that is orthogonal to ATP-dependent, *SEC* gene-requiring internalization of larger aryl cationic amphipaths (e.g., lysophospholipids), i.e., does not depend on the endocytic pathway [31]. However, passively internalized sertraline appears to be a potent substrate for ATP-dependent efflux pumps. In fact, efflux may explain the paradoxical observation that at physiological pH, sertraline is predicted to be over 99% ionized yet only a minority fraction (10–15%) is observed to be soluble at equilibrium. Our interpretation is that the ionized pool of sertraline is actively depleted both by ATP-dependent efflux *and* by sequestration in cellular membranes as a neutral species.

Despite our best efforts to separate vesiculogenic membranes into discrete organelles by density-gradient centrifugation, parsimony dictates that sertraline asymmetrically accumulates in the membranes of all V-ATPase-acidified organelles, presumably in proportion to local lysosomotropic driving forces. Specifically, sertraline associates with organellar membranes by two mechanisms: adsorption to solvent-exposed anionic sites, and intercalation into the bulk hydrophobic phase of the bilayer. A two-state, weakly binding and strongly binding model has been proposed for local anesthetic association with reconstituted liposomes [32], and we argue that adsorptive binding by sertraline in yeast cells may be mediated by electrostatic interactions, while intercalative binding by sertraline in yeast cells may be mediated by lipophilic interactions. However, several uncertainties remain in part because *in vivo* experiments on CAD in living cells are unlike experiments on CAD in reconstituted liposomes, in which the ionization state of CAD can be experimentally manipulated. Therefore, we conclude that sertraline-membrane association is a composite of more than one binding interaction, a conclusion supported by molecular dynamic simulations [33], [34].

The central finding of our study is that at micromolar doses, cellular membrane accumulation by sertraline induced curvature stresses throughout the organelles of the vesicular transport pathway. This membrane curvature stress triggers an autophagy-dependent membrane quality control response that appears to be enhanced in mutants with altered clathrin function. Although we have not provided biochemical evidence of autophagy induction, our ultrastructural approach revealed unambiguous induction of autophagy. We argue that autophagy mitigates cationic amphipath accumulation in cellular membranes. This interpretation is supported by a recent study that documented induction of autophagy by antidepressants in mammalian neuronal cell lines [35]. Autophagy may be one of several buffering systems that evolved to preserve cellular membrane homeostasis in the face of endogenous (or exogenous) charged amphipath accumulation, but when sertraline-induced membrane curvature stresses become too punishing at high or sustained doses, cytotoxicity ensues [36]. However, the cell-physiological effects of sub-lethal doses of sertraline may not be deleterious. We previously showed that low micromolar sertraline partially rescues the constitutive growth defect of yeast mutants with altered clathrin function [12]. If these buffering systems are defective due to genetic (e.g., clathrin dysregulation) and/or environmental stressors – resulting in a maladaptive homeostatic set point – sub-lethal accumulation of amphipath may normalize this set point. A “trophic” or cytoprotective effect might ensue given the ancient coupling between membrane transport and cell growth [37]. A similar argument was proffered by researchers explaining the cytotoxic effects of the phenothiazine antipsychotic drug chlorpromazine in yeast cells [13]. In conclusion, our model supports the notion of a serotonin transporter-independent component of antidepressant pharmacology in humans, and appears to buttress the neurotrophic hypothesis of depression [38]. It is tempting to speculate that amphipath accumulation may, in specific contexts, provide a trophic signal through direct modulation of the physical properties of cellular membranes, perhaps resulting in vesicle formation, thereby exploiting (or “short-circuiting”) the aforementioned coupling between membrane transport and cell growth. Such a mechanism would be expected to unfold over long time scales given the vigilance of those buffering systems, and the large effective volume of cellular membranes of the mammalian brain.

## Materials and Methods

### Yeast strains and culture conditions

Standard growth conditions were YPD media (1% yeast extract, 2% peptone and 2% dextrose) buffered with 10 mM HEPES to the desired pH. Some experiments with prototrophic strains were carried out in HEPES-buffered minimal media (yeast nitrogen base containing ammonium sulfate, 2% dextrose). BY4716, BY4742 or ACY769 were employed as wildtype reference strains as appropriate, and are all derived from S288c. sert^R^ mutant strains were previously described [12]. sert^HS^ deletion strains were derived from ACY769 and belong to a prototrophic homozygous deletion collection generated by D. Hess (Santa Clara University) and A. Caudy (University of Toronto). The *dnf1Δ dnf2Δ dnf3*Δ mutant was kindly provided by T. Graham (Vanderbilt). The *sec18^ts^* mutant was kindly provided by W. Prinz (NIH).

### Chemical compounds

Sertraline hydrochloride (Sigma-Aldrich) was resuspended in dimethyl sulfoxide (DMSO) to a final concentration of 25 mg/mL (∼73 mM), and 100 µL aliquots were stored in glass vials at −20°C until use and subjected to a maximum of one freeze/thaw cycle. Sertraline [N-methyl-^3^H] hydrochloride (American Radiolabled Chemicals, Inc.) is 99% pure by HPLC, has a specific activity of 80 Ci/mmol, and was kept at a stock concentration of 1 mCi/mL in ethanol.

### Pharmacological/biochemical assays and analysis

Overnight cultures were diluted in fresh pH-buffered YPD medium and incubated at 30°C till log phase. Cell number and cell size were determined by the Coulter counter method. For uptake and accumulation experiments, 0.25 µCi ^3^H-sertraline (American Radiolabeled Chemicals) was added to 2 mL yeast culture aliquot (all 0-minute time point aliquots contained 1 µM sodium azide and sodium fluoride). Cells were collected on Durapore* PVDF filters (Millipore) by passing through a filter unit, and washed with 18 ml ice cold pH-buffered YPD. Filters were incubated with 300 µL cold lysis buffer (acetonitrile∶methanol∶water, 40∶40∶20) at −20°C for 10 minutes. Cell pellets were collected by pipetting and transferred to scintillation vials containing 4 mL Cytoscint fluid (Fisher Scientific, Inc.). Counts were obtained using a Perkin Elmer Tri-Carb 2800TR liquid scintillation analyzer. For kinetic experiments, cells were incubated with specified concentrations of [^3^H]sertraline for 2 minutes, then collected on filters and washed with ice cold YPD containing 10 µM unlabeled sertraline [39]. Subcellular fractionation was carried out as follows. Cells were sphereplasted following Zymolyase (Zymo Research) digestion at 30°C for 1 hour according to manufacturers protocol. Spheroplasts were osmotically lysed in lysis buffer (50 mM Tris-HCl, 0.8 M sorbitol). The resulting lysate was centrifuged at 400 rcf for 10 minutes, yielding a post-nuclear lysate. An initial spin at 10,000 rcf for 10 minutes yielded P_10,000_ (enriched in large organellar membranes) and S_10,000_ fractions; S_10,000_ was spun at 100,000 rcf for 50 minutes, yielding a microsomal (P_100,000_) and a true soluble fraction (S_100,000_). All pellets were resuspended in equal volumes of the same buffer as the corresponding supernatant fractions. For experiments involving density-gradient centrifugation, the 10,000 rcf pellet obtained during fractionation was resuspended in 1 mL 35% Optiprep solution (Sigma Aldrich), on top of which 1 mL 30% Optiprep solution and 1 mL base solution (10 mM Tris-HCl pH 7.4, 150 mM NaCl, 8% sucrose) were layered consecutively. The gradient was centrifuged at 61,000 rpm for 17 hours and 45 minutes using a TLA100.3 rotor (Beckman). Ten equal fractions were collected from each gradient. Optiprep gradient fractions were pelleted with TCA, washed twice with ice-cold acetone, and resuspended in 30 µL Nu-PAGE SDS sample buffer (Invitrogen). Primary antibodies were used at the following dilutions: the late-Golgi marker Vsp10p 1∶250 (A-21274, Invitrogen); the vacuolar membrane marker ALP 1∶500 (A-6458, Invitrogen); the endoplasmic reticulum marker Dpm1p 1∶2000 (A-6429, Invitrogen); the endosomal marker Pep12p 1∶2000 (A-21273, Invitrogen); the mitochondrial marker Porin 1∶2000 (A-6449, Invitrogen); carboxypeptidase Y (ab113685, Abcam); and the plasma membrane marker Pma1p 1∶10,000 (ab4645, Abcam). For immunoblotting, total protein was transferred to PVDF membrane after electrophoresis using the iBlot system (Invitrogen). PVDF membranes were washed with SNAP I.D. Protein Detection System (Millipore), and chemiluminescence was detected using ECL plus Western Blotting Substrate (Pierce). Analysis of pharmacological data and figure generation were performed using Prism 5 (GraphPad Software, Inc).

### Transmission electron microscopy and analysis

We based our protocol on a membrane-preserving procedure previously described [40]. Our step-by-step protocol is available as **Methods S1**. 10 mL of fresh YPD media were inoculated with cells from an overnight culture and allowed to double several times to a maximum OD_600_ of ∼0.5. For drug treatments, cells were treated with 60 µM sertraline for 5, 10, 20 and 45 minutes. Multi-cell and single-cell fields were collected. Quantification of ultrastructural phenotypes was performed on a dataset compiled from wide fields as previously described [12]. Wide-field cell counts for the 45-minute dataset were as follows: BY4716 (untreated n = 216, sertraline-treated n = 193); prototroph (untreated n = 247, sertraline-treated n = 184); *chc1* (untreated n = 234, sertraline-treated n = 202); *swa2* (untreated n = 165, sertraline-treated n = 168); *vma9* (untreated n = 212, sertraline-treated n = 166); *sac1*Δ (untreated n = 219, sertraline-treated n = 151); *drs2*Δ (untreated n = 220, sertraline-treated n = 158); *arf1*Δ (untreated n = 194, sertraline-treated n = 133).

## Supporting Information

Figure S1 **Growth rate of BY4716 cells as a function of sertraline concentration.** Cells at optical density (OD_600_) equal to 1.0 were used as the initial inoculum.
(TIFF)

Methods S1 **Detailed protocol describing “ROTO” (Reduced Osmium tetroxide Thiocarbohydrazide - reduced Osmium) transmission electron microscopy.**
(DOCX)
