# Supplementary figures and images for: Accumulation of an Antidepressant in Vesiculogenic Membranes of Yeast Cells Triggers Autophagy
Source: PLoS One. 2012 Apr 18;7(4):e34024. doi: 10.1371/journal.pone.0034024 (PMC3329523; doi:10.1371/journal.pone.0034024)

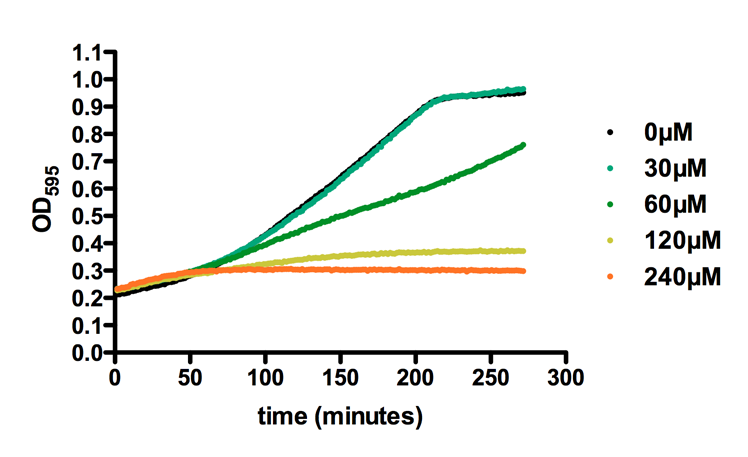

Supplement: Figure S1 — Growth rate of BY4716 cells as a function of sertraline concentration. Cells at optical density (OD600) equal to 1.0 were used as the initial inoculum. (TIFF) [file pone.0034024.s001.tiff]
