# Supplementary material for: Accumulation of an Antidepressant in Vesiculogenic Membranes of Yeast Cells Triggers Autophagy
Source: PLoS One. 2012 Apr 18;7(4):e34024. doi: 10.1371/journal.pone.0034024 (PMC3329523; doi:10.1371/journal.pone.0034024)
Supplement: Methods S1 — Detailed protocol describing “ROTO” (Reduced Osmium tetroxide Thiocarbohydrazide - reduced Osmium) transmission electron microscopy. (DOCX) [file pone.0034024.s002.docx]

**Methods S1**

Reagents:

Buffer A: 0.1M Cacodylate

5mM CaCl2

10 ml

Fix: Buffer A 5ml (2x stock)

3% glutaraldehyde 3ml (10%)

ddH2O 2ml

25ml 10ml

Wash buffer 1: 100mM Tris pH 8.0 2.5ml (1M) 1ml

25mM DTT .625ml (1M) .250ml

5mM EDTA .25ml (.5M) .100ml

1.2M sorbitol 12.5ml (2.4M) 5ml

ddH2O 9.125 3.65ml

Spheroplast buffer 0.1M potassium phosphate (pH 5.8)

1.2M sorbitol

For 8 ml

rOsO4 1% OsO4 2ml (4%)

1% K- ferrocyanide 2ml (4%)

Buffer A 4ml

1% Thiocarbohydrazide (TCH) dissolve at least 2 hours at 60C in water.

Fixation:

1. Inoculate 10 mL media from overnight culture to OD_600_ 0.2-0.3 and allow to grow up to OD_600_ 0.5-1.0.
2. Collect cells on filters and resuspend cells in 1mL Fix buffer.
3. Transfer into a new micro-centrifuge tube, incubate for 10 min and spin down. Add 500µl Fix and incubate for 2hrs (total). (Samples can be stored overnight at 4˚C overnight).
4. Wash cells in wash buffer. Resuspend in fresh wash buffer and incubate for 10 min.
5. Wash cells in Spheroplast buffer. Spin down and resuspend in 300µL of fresh Spheroplast buffer and add 12µL zymolyase. Incubate for 2 hrs at 30C.
6. Spin cells down and wash in buffer A.
7. Resuspend cells in 500ul 1% rOsO4 and incubate for exactly 30 min.
8. Wash 4 times with ddH2O
9. Resuspend cells in 1% TCH and incubate for exactly 5 min.
10. Wash 4 times with ddH2O
11. Resuspend cells in 1% rOsO4 and incubate for exactly 3 min.
12. Wash 4 times in ddH2O.

Dehydration and imbedding:

1. Dehydrate on ice
   1. 30% EtOH 5 min
   2. 50% EtOH 5 min
   3. 70% EtOH 5 min
   4. 70% EtOH 5 min
   5. 95% EtOH 5 min
   6. 100% EtOH 5 min
   7. 100% EtOH 5 min
   8. 100% EtOH 5 min
2. Imbed in LR white by washing cells with 1:1 LR white EtOH mix then incubating for 3 hour in 1:1 mix.
3. Spin and resuspend in 100% LR white to for 1hr. Spin down and resuspend in fresh LR white and incubate over night at 4˚C.
4. Resuspend cells in 600 fresh LR white and transfer to beem capsule, close and incubate at 60˚C overnight.

Cells were imbedded in LR White resin (Electron Microscopy Services). Samples were sectioned to 70µM with a microtome (Leica EMUC 6) and mounted on 300 mesh formvar carbon coated nickel grids (Electron Microscopy Services). Samples were viewed on an electron microscope (LEO 912AB with OMEGA ENERGY FILTER).
